# Targeted sequencing with enrichment PCR: a novel diagnostic method for the detection of EGFR mutations

**DOI:** 10.18632/oncotarget.3807

**Published:** 2015-04-12

**Authors:** Suki Kang, Baek Gil Kim, Hyun Ho Han, Joo Hyun Lee, Ji Eun Kim, Hyo Sup Shim, Nam Hoon Cho

**Affiliations:** ^1^ Department of Pathology, Yonsei University College of Medicine, Seoul, Korea; ^2^ Severance Biomedical Science, Yonsei University College of Medicine, Seoul, Korea; ^3^ Brain Korea 21 PLUS Project for Medical Science, Yonsei University, Seoul, Korea

**Keywords:** Enrichment ultra-deep pyrosequencing, EGFR mutation, lung cancer, diagnostic technique

## Abstract

Epidermal growth factor receptor (EGFR) is an important mediator of tumor cell survival and proliferation. The detection of EGFR mutations can predict prognoses and indicate when treatment with EGFR tyrosine kinase inhibitors should be used. As such, the development of highly sensitive methods for detecting EGFR mutations is important. Targeted next-generation sequencing is an effective method for diagnosing mutations. We compared the abilities of enrichment PCR followed by ultra-deep pyrosequencing (UDP), UDP alone, and PNA-mediated RT-PCR clamping to detect low-frequency EGFR mutations in tumor cell lines and tissue samples. Using enrichment PCR-UDP, we were able to detect the E19del and L858R mutations at minimum frequencies of 0.01% and 0.05%, respectively, in the PC-9 and H197 tumor cell lines. We also confirmed the sensitivity of detecting the E19del mutation by performing a titration analysis in FFPE tumor samples. The lowest mutation frequency detected was 0.0692% in tissue samples. EGFR mutations with frequencies as low as 0.01% were detected using enrichment PCR-UDP, suggesting that this method is a valuable tool for detecting rare mutations, especially in scarce tissue samples or those with small quantities of DNA.

## INTRODUCTION

Epidermal growth factor receptor (EGFR) is a critical mediator of tumor cell survival and proliferation [1]. *EGFR* is overexpressed in 43-89% of non–small-cell lung carcinoma (NSCLC) cells and has become an important therapeutic target for the treatment of lung cancer [2-5]. Mutations in this gene can predict prognoses and indicate the optimal timing for treatment with EGFR tyrosine kinase inhibitors (TKIs) [6, 7]. Therefore, the development of sensitive and specific methods for the detection of *EGFR* mutations would be valuable. Recent studies have attempted to develop such methods using Sanger sequencing, pyrosequencing, and specific real-time polymerase chain reaction (PCR) [4, 8, 9]. Although Sanger sequencing is considered as the gold standard for the detection mutations, this approach is limited by its low sensitivity and its requirement that mutant alleles exist at frequencies of at least 15-20% [10]. Ultra-deep pyrosequencing (UDP) overcomes some of these limitations by enabling amplification of the target DNA through PCR and by its capability to perform much longer reads than other techniques. In fact, this method often produces more than 10,000 reads per sequencing reaction [11, 12]. Despite the advantages that UDP technology offers over Sanger sequencing and PCR-based methods, UDP is still limited by its low sensitivity when screening for rare mutations [13-16]. Many efforts have been made to identify low-frequency genetic mutations that appear in approximately 2-5% of tumor cells using UDP technology [13, 17].

In this study, we compared three methods, namely peptide nucleic acid (PNA)–mediated PCR clamping, UDP, and enrichment PCR-UDP, to develop a more sensitive method for the detection of *EGFR* mutations. Here, we report that enrichment PCR-UDP can detect *EGFR* mutations with frequencies as low as 0.01% in heterogeneous samples. Our results can be used to assist in the identification of *EGFR* mutations in rare or difficult-to-obtain tissue samples.

## RESULTS

### Comparison of enrichment PCR-UDP, UDP, and PNA-mediated RT-PCR clamping

**Figure 1:**
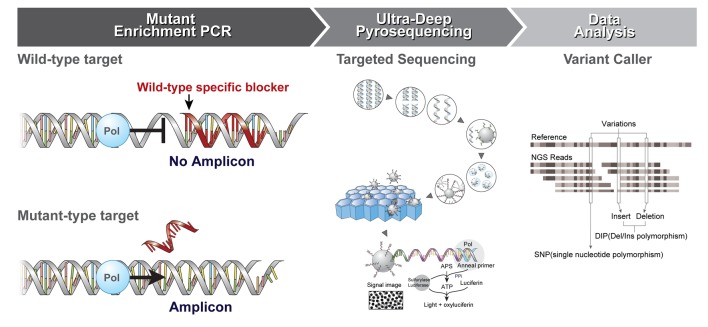
Schematic diagram of the enrichment PCR-UDP workflow Step I. Mutant Enrichment PCR. Step I. The wildtype-specific blocker suppresses amplification of the wildtype allele, which enables enrichment of the mutant allele. PCR amplification is conducted on the wildtype specific blocker (PNA probe, red). The blocking probe preferentially hybridizes to wildtype alleles and inhibits their amplification at the extension temperature (68°C), resulting in enrichment of mutant PCR fragments. Step II. Ultra-Deep Pyrosequencing (UDP): Sequencing library preparation PCR was performed using enrichment PCR products as a target and adaptor and barcode-conjugated primer pairs. PCR amplicons are analyzed by UDP as follows: sequencing library preparation PCR → library of single-stranded DNA fragments → one DNA molecule per bead → clonal amplification of DNA in emulsion → beads deposited into wells → independent sequencing of each bead [29]. Step III. Data Analysis: Variations can be detected by changing the number of sequence reads compared against a reference [22]. Pol, polymerase; APS, adenosine phosphosulfate; PPi, pyrophosphate; ATP, adenosine triphosphate

**Figure 2:**
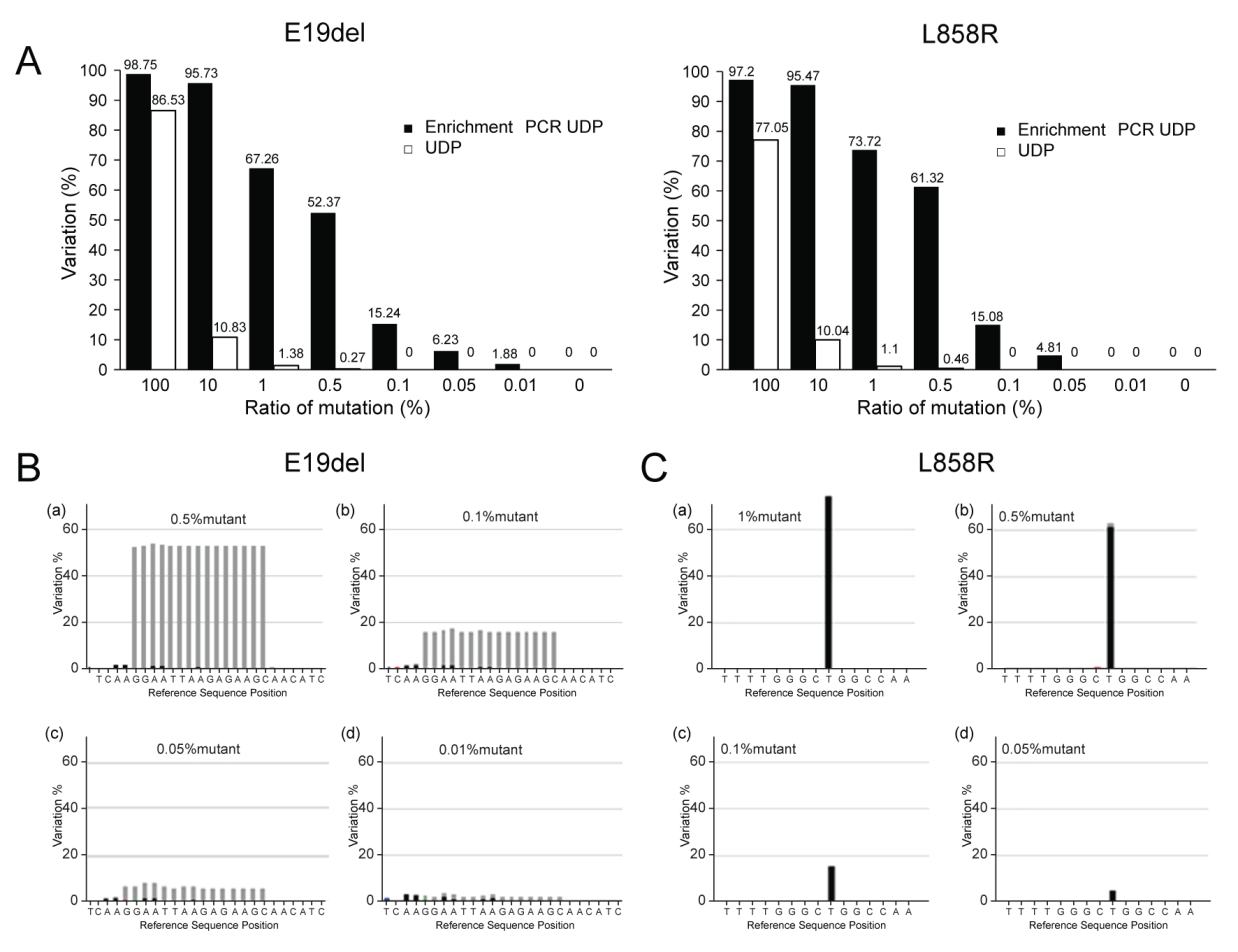
Comparison of enrichment PCR-UDP and UDP for detection of E19del and L858R *EGFR* mutations **A**. The horizontal axis indicates the ratio of *EGFR* mutation based on genomic DNA from PC-9 (left) and H1975 (right) cells mixed with HeLa cell genomic DNA. The vertical axis shows the observed variation results by enrichment UDP. **B**. A variation plot analysis of E19del. *EGFR* exon 19 deletions are in-frame deletions that occur within exon 19. **C**. A variation plot analysis of L858R (T>G). The L858R mutation results in a leucine to arginine substitution at position 858 of EGFR [27]. B and C, light gray: deletion; dark gray: point mutation.

We selected two lung cancer cell lines that exhibit mutations in *EGFR* to confirm the respective sensitivities of the UDP and enrichment PCR-UDP methods, namely PC-9 cells, which possess a deletion in exon 19 (E19del), and H197 cells, which contain a substitution mutation (L858R) in exon 21. Titration analysis using a mixture of HeLa and *EGFR* mutant cells was performed to evaluate the lower limit of detection for each method. The samples evaluated consisted of mixed populations of 100% (no HeLa cells), 10%, 1%, 0.5%, 0.1%, 0.05%, and 0.01% mutant cells (with either E19del or L858R), as well as HeLa cells alone. We analyzed serially diluted genomic DNA to obtain mutation/wild-type DNA proportions of 0, 0.01, 0.05, 0.1, 0.5, 1, 10, and 100%. Using enrichment PCR-UDP (Figure 1), we were able to detect the E19del and L858R mutations at minimum frequencies of 0.01 and 0.05%, respectively. However, the minimum frequency detected by UDP was only 0.5% (Figure 2). Thus, enrichment PCR-UDP was more sensitive than UDP in detecting low-frequency *EGFR* mutations.

Next, we tested whether enrichment PCR-UDP and UDP can detect rare *EGFR* mutations within the same FFPE tumor tissues, and correlated these results with those obtained in the same tissues by PNA-mediated real-time PCR clamping. Enrichment PCR-UDP detected 90.35% variation for E19del in FFPE sample 1 with a higher resolution, whereas UDP detected 1.73% variation for E19del in the same sample. Moreover, the resolution of the variation ratio of L858R in samples 7 and 8 was increased in enrichment PCR-UDP (25.29-99.29% and 3.8-88.83% in FFPE samples 7 and 8, respectively (Table 1). To assess the possibility of false-positive or false-negative results, we screened the FFPE samples for both mutations using PNA-mediated real-time PCR clamping. These results were identical to those from enrichment PCR-UDP, indicating the lack of false-positive or false-negative results from the enrichment PCR-UDP approach. Tables S1 and S2 summarize the number of passed reads, total length of the sequencing data, and average read length at each base across all runs for the sequencing library pools and the barcoded amplicon runs.

**Table 1:** Comparison of PNA-mediated PCR clamping, UDP, and enrichment PCR-UDP in detecting *EGFR* mutations in FFPE samples

Mutation	Samples	PNA-mediated real-time PCR clamping	Ultra-deep pyrosequencing		Enrichment PCR ultra-deep pyrosequencing	
			Variation(%)	Depth	Variation(%)	Depth
E19Del	FFPE S1	E19del	1.73	7670	90.53	15286
	FFPE S2	WT	0	17715	0	26976
	FFPE S3	WT	0	13677	0	9867
	FFPE S4	WT	0	16526	0	51322
	FFPE S5	WT	0	16143	0	20723
	FFPE S6	WT	0	20098	0	14197
L858R	FFPE S7	L858R	25.29	4225	99.29	5091
	FFPE S8	L858R	3.8	3921	88.83	3481
	FFPE S9	WT	0	6112	0	4733
	FFPE S10	WT	0	5730	0	4823

### Confirmation of the sensitivity of detection in FFPE samples

**Figure 3:**
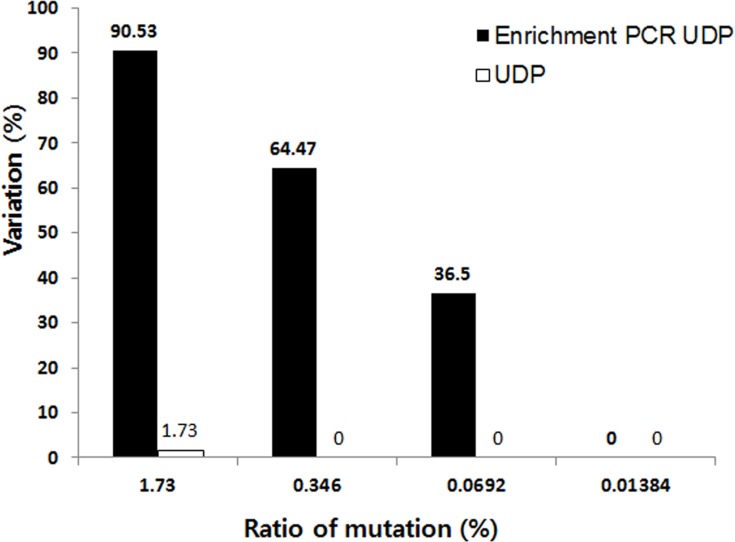
The sensitivity of mutation detection by UDP and enrichment PCR-UDP in FFPE samples The horizontal axis shows the expected value of the fraction of *EGFR* E19del mutants in the FFPE sample. The vertical axis shows the observed variation results of *EGFR* E19del mutants by UDP and enrichment PCR-UDP.

We confirmed the sensitivity of E19del detection by performing a titration analysis on FFPE sample 1. Sample DNA was serially diluted to produce mutation frequencies of 1.73, 0.346, 0.0692, and 0.01384%. Using enrichment PCR-UDP, the lowest mutation frequency detected was 0.0692%. In contrast, the lowest mutation frequency detected by the UDP method was 1.73% (Figure 3). Thus, we confirmed that enrichment PCR-UDP is more sensitive than UDP for the detection of *EGFR* mutations in FFPE samples.

## DISCUSSION

New methods for the detection of low-frequency mutations in heterogeneous samples are needed to improve diagnostic accuracy and, ultimately, patient prognosis. In this study, we evaluated the use of the newly developed enrichment PCR-UDP method in enhancing the detection of mutations via parallel pyrosequencing with the Roche 454 Junior system (Figure 1). We found that PCR enrichment combined with UDP could detect lower-frequency *EGFR* mutations than UDP alone. The copy number of mutated sequences was magnified by PCR enrichment before next generation sequencing (NGS)-based amplicon resequencing, which enabled a clear distinction between actual mutations and background sequencing noise. Combining blocker-PCR technology with UDP improved the detection limit of targeted amplicon resequencing of low-abundance mutations. Enrichment PCR-UDP is advantageous due to its high sensitivity in detecting mutant alleles and single nucleotide polymorphism variants. Moreover, we selected PNAs for use as a blocker with UDP because these molecules form a polyamide skeleton that is not affected by salt concentration and maintains a stable bond [18]. The thermal stability of PNA binding to complementary nucleic acids is higher than that of DNA and RNA. Because they are not substrates for DNA polymerases, PNA oligomers suppress the amplification of wild-type sequences confined by pairs of DNA oligonucleotide primers in PNA-mediated enrichment PCR [19].

In this study, we compared three methods for the detection of *EGFR* mutations, namely PNA-mediated PCR clamping, UDP, and enrichment PCR-UDP. Both UDP alone and enrichment-based UDP methods utilize the same PCR primer pairs that contain barcodes, sequencing adaptors, and the PCR premix. The only major difference is whether or not wild-type blocker (PNA probe) is incorporated. These advances are derived from mutant-specific amplification methods that make use of a polymerase elongation arrest strategy [20]. Competitive clamping was more effective than elongation arrest. However, compared to competitive clamping, clamping by elongation arrest offers greater flexibility in the choice of target sites for PNA oligomers and primers. This flexibility facilitates NGS sequencing panel design with mutant-enriched sequencing results. In the competition method, primer design is restricted by position and temperature. Moreover, the primers must avoid other possible mutations, making it more difficult to use in NGS systems. A PNA clamping-based method uses “competition of primer and PNA probe” for mutant-specific PCR. In our approach, we are taking advantage of the benefits associated with the “elongation arrest method” and “blocking of polymerase extension”.

Enrichment PCR-UDP technology is integrated NGS technology that is based on UDP but incorporates PNA blocking to increase the sensitivity of mutation detection. After mutation-specific PCR amplification, samples are prepared for NGS analysis by removing nonspecific amplification products. Recent studies reported the development of various technologies, including targeted NGS, PNA-mediated PCR clamping, and allele-specific PCR, that enable greater sensitivity for the detection of minor mutant alleles with low frequencies [21-23]. Moreover, various technologies have been developed and tested for the detection of *EGFR* mutations. DeBiase et al. compared UDP with Sanger sequencing and demonstrated that Sanger sequencing was incapable of detecting mutations below 40%, whereas NGS detected a proportion of neoplastic cells as low as 5% [24]. Bellevicine et al. reported an immunocytochemistry-based method using mutant-specific anti-EGFR antibodies that detected 10% mutation [25]. Comparison of the amplification refractory mutation system (ARMS) with Sanger sequencing by Shaozhang et al. revealed that ARMS (94.4%) was more sensitive than Sanger sequencing (72.2%) for the detection of *EGFR* mutations in patients with NSCLC [26]. However, the lack of a recognized standard method renders the evaluation of these technologies difficult. We also tested *EGFR* mutations using a PNA-mediated PCR clamping method to determine the frequency of *EGFR* mutations in heterogeneous samples. This highly sensitive method has been demonstrated to detect *EGFR* mutations in the presence of background signals that are 100 to 1,000 times more abundant [23, 27]. Our results showed that enrichment PCR-UDP produces results similar to PNA-mediated real-time PCR clamping for the detection of *EGFR* mutations. Therefore, enrichment PCR-UDP is a sensitive method that can be used to detect rare mutations, and is a viable alternative to PNA-mediated real-time PCR clamping (allele-specific real-time PCRs).

Although FFPE tissue samples can offer significant patient information, the quantity and quality of the tissue contained within them are often inadequate for detecting rare mutant alleles. Hence, highly sensitive methods are required to detect mutant alleles in FFPE samples. We demonstrated the performance of enrichment PCR-UDP in the detection of rare *EGFR* mutations in FFPE samples. In addition, we established and validated its use in the detection of E19del and L858R mutations in FFPE samples, which contain small amounts of DNA. In fact, our sensitivity analysis demonstrated that up to 5 ng of DNA was available for analysis from each of our tissue samples, and that enrichment UDP can detect low-frequency genetic mutations (<0.05%).

Enrichment PCR-UDP is a powerful method for NGS that, when combined with ongoing advances in the detection and quantitation of *EGFR* mutations, will help to better identify patients with NSCLC, who are most likely to derive the greatest benefit from treatment with EGFR TKIs. Drug-sensitive *EGFR* mutations are reported in 10-30% of patients with NSCLC [28]. Thus, detection of genetic changes in *EGFR* is critical for improving diagnosis and developing targeted therapies. Enrichment PCR-UDP can target any region of interest with a high level of sensitivity and specificity easily and rapidly. Therefore, this novel technology may become one of the most practical and useful methods for the detection of *EGFR* mutations in NSCLC patients. Moreover, this approach will aid in the development of additional technologies for the detection of rare *EGFR* mutations in clinical trials.

## MATERIALS AND METHODS

### Preparation of genomic DNA targets

Genomic DNA from PC-9 cells, which harbor a E19del deletion in exon 19, and H1975 cells, which possess an L858R mutation in exon 21 of *EGFR*, were serially diluted to the ratios of 0, 0.01, 0.05, 0.1, 0.5, 1, 10, and 100% with HeLa cell genomic DNA (New England Biolabs, Beverly, MA, USA) to a final concentration of 15 ng/μL. Genomic DNA was extracted from cells using the High Pure PCR Template Preparation Kit (Roche Diagnostics, Mannheim, Germany) according to the manufacturer's instructions. The manufactured DNA targets were stored at −20°C until use. The clinical formalin-fixed paraffin-embedded (FFPE) tumor samples were obtained from Yonsei Medical University Hospital. This study was approved by the Hospital Ethics Committee.

### PNA-mediated EGFR real-time PCR clamping

The *EGFR* real-time PCR clamping mutation kit (Panagene Inc., Daejeon, Korea) was used according to the manufacturer's instructions. Real-time PCR was performed with a real-time PCR system (CFX 96, Bio-Rad, Hercules, CA, USA) using 15 ng of genomic DNA. The data for each mutation were interpreted according to the kit manual after curve analysis and calculation of ΔCt values.

### PCR amplification for conventional next-generation sequencing

The target samples were analyzed by next-generation sequencing (NGS) for *EGFR* mutations with the Cancer Panel on a GS Junior Sequencer (Roche Diagnostics). For conventional 454-targeted resequencing, 30 ng of genomic DNA was used in the PCR of the *EGFR* panel (SeaSun Biomaterials, Daejeon, Korea). Subsequent processing of the samples was performed according to the manufacturer's protocol.

### Enrichment PCR for mutant-enriched NGS and sequencing library preparation

To increase the resolution of low-level somatic mutant molecules within a high background of wildtype molecules, the Insight^TM^ Onco Panel for *EGFR* (SeaSun Biomaterials) was used for mutant enrichment PCR according to the manufacturer's instructions. This assay was performed using 30 ng of genomic DNA, and subsequent processing of the samples was performed according to the manufacturer's protocol. PCR samples contained non-specific PCR products that mainly included primer dimers, which make NGS sequencing difficult due to their short read lengths. These non-specific PCR products were removed by AgencourtAMPure XP beads (Beckman Coulter, Vienna, Austria) using a 1:1 DNA-to-bead ratio. Sequencing library preparation PCR was performed using 2 μL of purified PCR product from enrichment PCR amplification as a template, *EGFR* Insight 2x Seq Lib Pep Premix (SeaSun Biomaterials), and each barcoded primer pair. The sequencing adaptor with a multiplex identifier was conjugated using the manufacturer's protocol. Any unwanted short fragments were removed with AgencourtAMPure XP beads (Beckman Coulter) using a 1:1 DNA-to-bead ratio.

### Quantitation and normalization of sequencing amplicons

The purified amplicons were quantitated by Pico-Green (Life Technologies, Carlsbad, CA, USA) utilizing an external Infinite F200Pro fluorometer (Tecan, Grodig, Austria) with Magellan v7.0 Software (Tecan). Based on the standard concentrations, the signals were directly converted to ng/μL, and the coefficient of determination (validation criteria, r^2^ > 0.99) was calculated from eight DNA standards ranging from 0 to 100 ng/μL. For emulsion PCR amplification, the concentrations of the amplicons were converted to molecules per μL using the associated amplicon length. The manufactured DNA pools were stored at −20°C until further use.

### Ultra-deep pyrosequencing

Pyrosequencing of the amplicons was performed according to the manufacturer's protocol using the GS Junior System (Roche Diagnostics). Emulsion PCR, breaking, and bead enrichment were conducted using the GS Junior Titanium emPCR Kit, Lib-L emPCR Reagents, Lib-L Kit, Oil and Breaking Kit, and the Bead Recovery Reagents Kit according to the supplier's instructions (Roche Diagnostics). For emulsion PCR, we used a copy-per-bead ratio of 0.5. Enrichment of the DNA-carrying magnetic beads was accomplished using a magnetic particle collector (Invitrogen, Life Technologies). The quantity of the enriched beads was determined with the GS Junior Bead Counter (Roche Diagnostics). Finally, we loaded 100,000 to 500,000 beads onto the PicoTiterPlate (Roche Diagnostics). Sequencing was carried out according to standard Roche/454 protocols using the GS Titanium Sequencing Kit (Roche Diagnostics) and the GS Junior device.

### Data analysis

Processed and quality-filtered reads were analyzed and the sequencing data were visualized using the GS Amplicon Variant Analyzer (Roche Diagnostics). Target amplicons (excluding adaptors and multiplex identifiers) were used as references to align the amplicon reads. The template-specific portions of the fusion primers were considered as primer A and primer B, and the known mutations in the selected samples were defined as substitutions relative to the reference sequence.

## SUPPLEMENTARY MATERIAL AND TABLES


