# Supplementary material for: Targeted sequencing with enrichment PCR: a novel diagnostic method for the detection of EGFR mutations
Source: Oncotarget. 2015 Apr 12;6(15):13742–9. doi: 10.18632/oncotarget.3807 (PMC4537046; doi:10.18632/oncotarget.3807)
Supplement: Supplementary file 1 [file oncotarget-06-13742-s001.pdf]

Targeted sequencing with enrichment PCR: a novel diagnostic method for the detection of EGFR mutations

Supplementary Material

Table S1: Sequencing library pools and barcoded amplicons.

Mutation	Sample		MID	Application	Library Pool
E19del	Cell line dilutant	100% (PC-9)	1	UDP/Enrichment PCR-UDP	A/B
		10%	2		
		1%	3		
		0.5%	4		
		0.1%	5		
		0.05%	6		
		0.01%	7		
		0% (HeLa)	8		
	Clinical tissue	FFPE S1	1	UDP/Enrichment PCR-UDP	C/D
		FFPE S2	2		
		FFPE S3	3		
		FFPE S4	4		
		FFPE S5	5		
		FFPE S6	6		
L858R	Cell line dilutant	100% (H1975)	1	UDP/Enrichment PCR UDP	E/F
		10%	2		
		1%	3		
		0.5%	4		
		0.1%	5		
		0.05%	6		
		0.01%	7		
		0% (HeLa)	8		
	Clinical tissue	FFPE S7	1/2	UDP/Enrichment PCR-UDP	G
		FFPE S8	3/4		
		FFPE S9	5/6		
		FFPE S10	7/8		

Table S2: Summary of sequencing results.

Library	Mutant	Target	Application	Total Bases	Passed Read	Passed %
A	E19del	PC-9 dilutant	UDP	23,787,201	108,588	79.87%
B			Enrichment PCR-UDP	18,247,209	94,679	88.90%
C		FFPE tissue	UDP	28,736,981	128,109	84.67%
D			Enrichment PCR-UDP	31,254,027	140,433	85.98%
E	L858R	H1975 dilutant	UDP	14,493,199	71,211	34.76%
F			Enrichment PCR-UDP	8,744,615	42,912	23.61%
G		FFPE tissue	UDP/Enrichment PCR-UDP	8,458,574	46,177	24.52%
